# LY75 Ablation Mediates Mesenchymal-Epithelial Transition (MET) in Epithelial Ovarian Cancer (EOC) Cells Associated with DNA Methylation Alterations and Suppression of the Wnt/β-Catenin Pathway

**DOI:** 10.3390/ijms21051848

**Published:** 2020-03-07

**Authors:** Sadia Mehdi, Magdalena Bachvarova, Marie-Pier Scott-Boyer, Arnaud Droit, Dimcho Bachvarov

**Affiliations:** 1 Department of Molecular Medicine, Université Laval, Québec, QC PQ G1V 0A6, Canada; sadia.mehdi.1@ulaval.ca (S.M.); Arnaud.Droit@crchudequebec.ulaval.ca (A.D.); 2 Research Center of Quebec CHU-Université Laval, Québec, QC PQ G1E6W2, Canada; magdalenab3@hotmail.com (M.B.); MariePier.ScottBoyer@crchudequebec.ulaval.ca (M.-P.S.-B.)

**Keywords:** epithelial ovarian cancer, epithelial–mesenchymal transition, reduced representation bisulfite sequencing, DNA methylation, LY75, Wnt/β-catenin

## Abstract

Growing evidence demonstrates that epithelial–mesenchymal transition (EMT) plays an important role in epithelial ovarian cancer (EOC) progression and spreading; however, its molecular mechanisms remain poorly defined. We have previously shown that the antigen receptor LY75 can modulate EOC cell phenotype and metastatic potential, as LY75 depletion directed mesenchymal–epithelial transition (MET) in EOC cell lines with mesenchymal phenotype. We used the LY75-mediated modulation of EMT as a model to investigate for DNA methylation changes during EMT in EOC cells, by applying the reduced representation bisulfite sequencing (RRBS) methodology. Numerous genes have displayed EMT-related DNA methylation patterns alterations in their promoter/exon regions. Ten selected genes, whose DNA methylation alterations were further confirmed by alternative methods, were further identified, some of which could represent new EOC biomarkers/therapeutic targets. Moreover, our methylation data were strongly indicative for the predominant implication of the Wnt/β-catenin pathway in the EMT-induced DNA methylation variations in EOC cells. Consecutive experiments, including alterations in the Wnt/β-catenin pathway activity in EOC cells with a specific inhibitor and the identification of LY75-interacting partners by a proteomic approach, were strongly indicative for the direct implication of the LY75 receptor in modulating the Wnt/β-catenin signaling in EOC cells.

## 1. Introduction

Epithelial ovarian cancer (EOC) accounts for 5% of all cancers in women and is the leading cause of death from gynecologic malignancies [1,2]. Despite treatment improvements, long-term survival rates for patients with advanced disease remain disappointing [3]. EOC lethality primarily stems from the inability to detect the disease at an early, organ-confined stage, and the lack of effective therapies for advanced-stage disease (e.g., metastasis) [4]. Indeed, despite advances in cytotoxic therapies [5,6], only 30% of patients with advanced-stage EOC survive 5 years after initial diagnosis [4]. One way to resolve this problem is to target metastasis-specific pathways with novel therapies. Hence, focused identification of novel prometastatic EOC pathways and molecules could improve the chances of discovering new and more effective EOC therapies [4].

Metastasis is a complex multistep process in the progression of cancer, causing approximately 90% of all human cancer mortalities [7]. To colonize a distant secondary site, cancer cells undergo epithelial–mesenchymal transition (EMT) characterized by the suppression of epithelial markers E-cadherin and EpCAM and acquisition of migratory capacity, pivotal for invasion and metastasis. Although EMT is clearly important to tumor progression, it is inconsistent with the observation that metastatic lesions mostly exhibit epithelial phenotypes, thus suggesting that mesenchymal–epithelial transition (MET) is critical to the latter stages of metastasis [7]. EMT has emerged as a key regulator of various biological processes implicated in embryogenesis, organ fibrosis, and cancer metastasis [8], including EOC dissemination [9,10,11]; however, the molecular mechanisms sustaining this process in EOC remain poorly defined. Both EMT and MET involve widespread reprogramming of gene expression and as recently shown, epigenetic mechanisms, that include post-translational histone modifications, noncoding RNAs (ncRNA), and DNA methylation could play important roles in these processes [12,13,14].

We have previously shown that the antigen receptor LY75 (also known as DEC205/CD205) can modulate EOC cell phenotype and metastatic potential [7]. Indeed, LY75 depletion directed MET in EOC cell lines with mesenchymal-like phenotype (SKOV3 and TOV112), associated with the induction of the expression of the epithelial markers E-cadherin, EpCAM, and EMP1 and loss of expression of the mesenchymal markers N-cadherin, TWIST1, FN1, and SNAIL1 [7]. Moreover, re-expression of a shRNA-resistant LY75 gene variant in the LY75 knockdown SKOV3 clones (SKOV3-shR) completely restored the initial mesenchymal phenotype and re-established the SKOV3 parental pattern of mesenchymal markers’ expression [7].

In the present study, we used the LY75-mediated modulation of EMT in EOC cells as a model to investigate DNA methylation changes during EMT in EOC cells. We applied the reduced representation bisulfite sequencing (RRBS) approach, a bisulfite-based cost-effective protocol that enriches CpG-dense regions of the genome, thus reducing the amount of sequencing required, while capturing the majority of promoters and other relevant genomic regions [15]. This approach led to the identification of numerous genes showing altered DNA methylation patterns following LY75-mediated EMT alterations in SKOV3 cells. Some of these genes could be implicated in EOC progression and/or could represent new EOC therapeutic targets. Consecutive Ingenuity Pathway Analysis (IPA) of the methylation data was strongly indicative for the predominant implication of the Wnt/β-catenin signaling pathway in the EMT-induced DNA methylation variations in EOC cells mediated by the LY75 expression changes. Consecutive experiments, including alterations in the Wnt/β-catenin signaling activity in EOC cells with the use of a specific inhibitor, and the identification of LY75-interacting partners by a proteomic approach, were strongly indicative for the direct role of LY75 in modulating the Wnt/β-catenin pathway activity in EOC cells.

## 2. Results

### 2.1. Reduced Representation Bisulfite Sequencing (RRBS) Analysis of Altered DNA Methylation Patterns during LY75-Mediated EMT in SKOV3 Cells; Identification of Novel Genes Displaying EMT-Associated DNA Methylation Variations

We applied the RRBS technology in order to identify specific genomic regions that undergo DNA methylation alterations during LY75-mediated EMT in EOC cells. Thus, we analyzed the DNA methylation patterns in previously generated SKOV3 cell clones with mesenchymal (M) phenotype (sh-control-SKOV3, SKOV3-shR), as compared to SKOV3 cell clones displaying epithelial (E) phenotype (sh-LY75-SKOV3, LY75-KO-SKOV3), as described before [7]; see also Table 1A for details. Three different experimental comparison (SKOV3-M vs. SKOV3-E) combinations were used for RRBS analysis, as shown in Table 1B.

**Figure 1:**
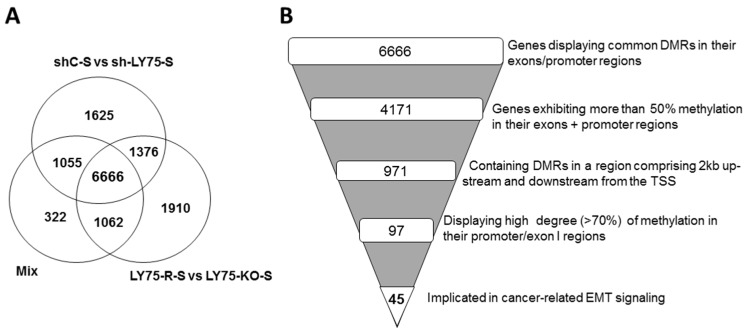
RRBS analysis of genes displaying common differentially methylated regions (DMRs) in their exons and promoter regions. (**A**) Venn diagram analysis of the three different comparison (M vs. E) experimental combinations used for RRBS analysis (see Table 1B for details); (**B**) A funnel plot indicating the selection criteria for the genes retained for further analyses, exhibiting a high degree of methylation in their promoter regions and implicated in cancer-related EMT signaling.

**Figure 2:**
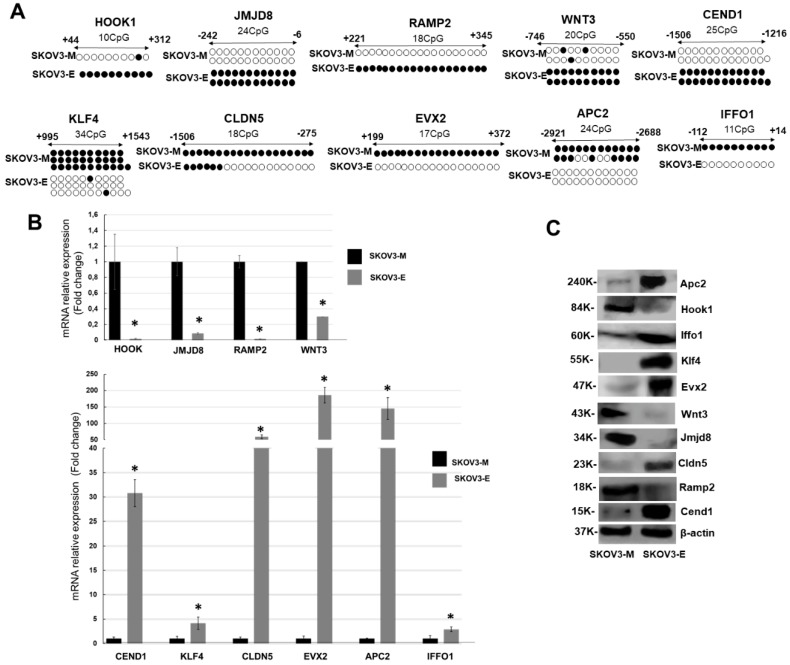
Validation of methylation status and the corresponding expression levels of the ten RRBS-retained genes in the SKOV3-LY75-related EMT model. (**A**) Bisulfite-sequencing PCR (BSP) analysis of the methylation status of selected genes in the SKOV3-M (sh-control) and the SKOV3-E (sh3-8 clone). Filled circles represent methylated CpGs and open circles represent unmethylated CpGs. The indicated positions on the CpG plots represent the number of nucleotides stretching upstream (+) and downstream (−) of the transcription initiation (ATG) codon for each gene analyzed. (**B**) Quantitative PCR analysis of the mRNA expression profiles of the ten selected genes in SKOV3-M and SKOV3-E cells. In all analyses, mRNA levels were displayed as relative to their expression levels in SKOV3-M cells and normalized to the ribosomal 18S (control) gene expression. Error bars represent SD; * *p* < 0.05. (**C**) Western blot analysis of the protein expression levels of the 10 selected genes in SKOV3-M and SKOV3-E cells. β-Actin was used as a loading control.

Further analysis of the sequencing data based on differentially methylated regions (DMRs) covering genes’ exons and promoter regions led to the identification of ~10,000 genes displaying DMRs for each of the three experimental combinations used (see Table 1B and Supplementary Table S1A–C). As shown in Figure 1A, consecutive Venn diagram analysis of the DMRs data from the three experimental combinations revealed 6666 genes, displaying common altered DMRs in their exons/promoter regions, following LY75-mediated EMT alterations in SKOV3 cells (see Supplementary Table S1D). Based on the 6666 gene list, we performed consecutive selections based on increasing-stringency criteria in order to retain highly hyper- or hypomethylated genes with potential role in EMT-mediated EOC dissemination (Figure 1B). Thus, we initially selected genes exhibiting more than 50% methylation in their exons/promoter regions (4171 genes), then we focused on genes displaying predominant methylation alterations in a region comprising 2 kb upstream and downstream from the transcription start site (TSS; 971 genes). Using the Integrative Genomic Viewer (IGV) software, we further selected genes with high degree (≥70%) of CpG island methylation at their promoter/exon I regions (97 genes), and finally retained 45 genes, shown previously to be implicated in cancer-related EMT signaling (see Supplementary Table S1E for the 45-gene list). Bisulfite-sequencing PCR (BSP) validation of DNA methylation status of most of the 45 genes, combined in parallel with analysis of their mRNA and protein expression levels, led to final selection of 10 genes, including HOOK1, RAMP1, JMJD8, and WNT3, hypermethylated in SKOV3-E cells, and CEND1, EVX2, CLDN5, APC2, IFFO1, and KLF4, hypomethylated in SKOV3-E cells (see Supplementary Table S1E for details). Figure 2A shows the BSP-mediated confirmation of the methylation status of these genes, which precisely correlated with both their mRNA (Figure 2B) and protein (Figure 2C) expression values in the corresponding SKOV3 cell clones.

These data were further confirmed upon performing shRNA-mediated LY75 knockdown in the serous EOC cell line OVCAR8, which also exhibits a mesenchymal-like phenotype. Indeed, the shRNA-mediated LY75 knockdown OVCAR8 clones sh-63 and sh-64 displayed a typical epithelial morphology (see Supplementary Figure S1A), accompanied with the overexpression of E-cadherin, and the suppression of N-cadherin, TWIST1, and SNAIL1 (Supplementary Figure S1B). As shown in Supplementary Figure S1C, the protein expression profiles of the 10 genes described above displayed quite similar expression patterns in OVCAR8-M (control) and OVCAR8-E (sh-63) cells as those found in SKOV3-E or SKOV3-M cells (see Figure 2C for comparison).

### 2.2. LY75-Mediated EMT Alterations in EOC Cells are Associated with Predominant Epigenetic Regulation of Members of the Wnt/β-Catenin Pathway

**Figure 3:**
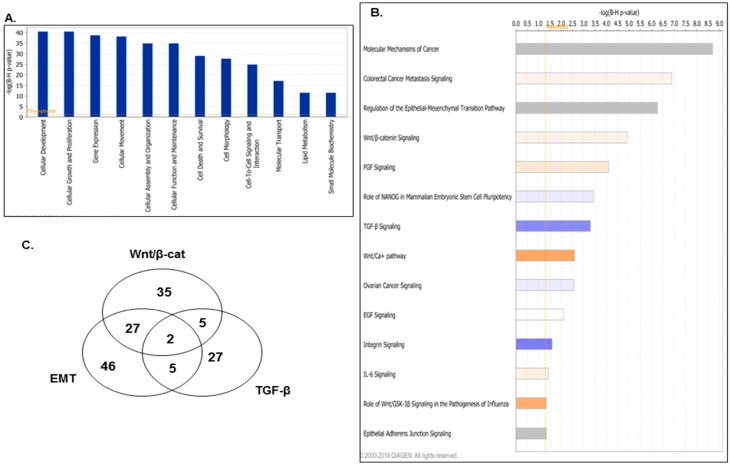
Ingenuity pathways analysis (IPA) of the 6666 common hyper- and hypomethylated genes in SKOV3-E cells. (**A**) Functional analysis for a dataset of the common hyper- and hypomethylated genes. Top functions that meet a Bonferroni–Holm multiple testing correction *p*-value of 0.05 are displayed. (**B**) List of selected canonical pathways that were significantly altered in SKOV3-E cells. Top functions that meet a Bonferroni–Holm multiple testing correction *p*-value of 0.05 are displayed. (**C**) Venn diagram analysis displaying common genes between the EMT, Wnt/β-catenin, and TGF-β signaling pathways, as derived from the IPA canonical pathways analysis.

We further analyzed the 6666 genes displaying common DNA methylation alterations in their exons/promoter regions upon LY75-mediated EMT by using the Ingenuity Pathways Analysis (IPA) software to identify relevant biological pathways and networks. As shown in Figure 3A, IPA analysis was indicative for EMT-related epigenetic alterations of functionally related groups, mostly linked to cellular development, cellular growth and proliferation, cellular movement, cellular function and maintenance, and importantly -cellular morphology. As expected, consecutive IPA canonical pathway analysis displayed predominant modulation of EMT-pathway-related genes and genes implicated in ovarian cancer signaling, as genes related to other major EMT-related pathways (including the Wnt/β-catenin, the Wnt/Ca^2+^, and the TGF-β pathways) similarly exhibited significant DNA methylation alterations (see Figure 3B and Table A1).

Remarkably, and as shown in Table A1, the number of Wnt/β-catenin-pathway-related genes with altered DMRs (69 genes) was significantly higher, compared to the TGF-β-pathway-related genes (39 genes). This was further supported by the number of the common genes shared between the EMT and Wnt/β-catenin pathways (27 genes) compared to those of the EMT/TGF-β pathways (five genes; see Figure 3C and Table A1), suggesting that the Wnt/β-catenin signaling might be the predominant pathway modulated by the LY75-mediated EMT alterations in EOC cells.

### 2.3. LY75 Expression Modulates the Wnt/β-Catenin Pathway Activity in EOC Cells

**Figure 4:**
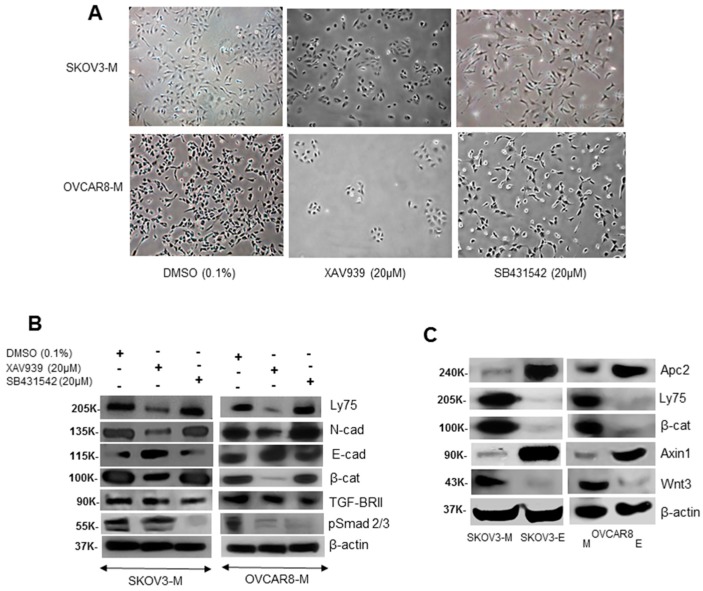
(**A**) Representative images of SKOV3-M and OVCAR8-M (parental) cells upon XAV939, SB431542, or DMSO (control) treatment. (**B**) Western blot analysis of Ly75, β-catenin, TGF-B-RII, p-Smad2/3, E-cadherin, and N-cadherin expression levels in SKOV3-M and OVCAR8-M cells upon treatment with the Wnt/β catenin inhibitor XAV939, the TGF-β inhibitor SB431542, and DMSO (control treatment). (**C**) Western blot analysis of the expression levels of different members (β-catenin, Axin1, Apc2, and Wnt3) of the Wnt/β catenin pathway in SKOV3-M, SKOV3-E, OVCAR8-M, and OVCAR8-E cells. β-Actin was used as a loading control.

The above IPA analyses prompted us to more profoundly investigate the relative implications of the Wnt/β-catenin and the TGF-β signaling pathways during the LY75-mediated EMT alterations in EOC cells. As shown in Figure 4A, treatment of SKOV3-M and OVCAR8-M (parental) cells with the Wnt/β-catenin inhibitor XAV939 led to the acquirement of epithelial-like cellular phenotype, while treatment with the TGF-β inhibitor SB431542 had no effect on SKOV3-M and OVCAR8-M cellular morphologies. XAV939 treatment resulted in N-cadherin protein suppression and E-cadherin protein overexpression in SKOV3-M and OVCA8-M cells, similar to the LY75 KD effect in these cell lines (Figure 4B). Moreover, XAV939 treatment in both these cell lines was also associated with decreased levels of β-catenin, while members of the TGF-β pathway, including TGF-βRII and the phosphorylated form of Smad2/3 (pSmad2/3) were not affected (Figure 4B). Interestingly, XAV939 treatment resulted in reduced LY75 expression in EOC cells, suggestive for a possible feed-back mechanism between Wnt/β-catenin signaling and LY75 functional activity (Figure 4B). The use of the TGF-β inhibitor SB431542 was only associated with pSmad2/3 suppression, especially in SKOV3-M cells; however, this inhibitor induced no changes in the N-cadherin and E-cadherin expression levels in both EOC cell lines, despite a prolonged (5 days) treatment (Figure 4B). The impact of the LY75 gene expression on the Wnt/β-catenin pathway was further confirmed by Western blot analysis of the expression levels of some of its members and regulatory proteins. Thus, LY75 KD in both SKOV3 and OVCAR8 cells was associated with decreased β-catenin and Wnt3 expression and strong induction of the expression of Axin1 and APC2, both previously characterized as members of the suppressor complex of the Wnt/β-catenin pathway ([16]; see Figure 4C). This was also confirmed by immunofluorescence analysis of Axin1 and APC2 expression in the SKOV3-E cells and high expression and nuclear localization of β-catenin in the SKOV3-M (control) cells (Supplementary Figure S2).

Moreover, analysis of the TCGA data for ovarian cancer via the cBioPortal software (https://www.cbioportal.org/) was indicative for significant LY75 co-expression correlations (*p* ≤ 0.05) with 5046 genes (Supplementary Table S2A), including most (64) of the Wnt/β-catenin pathway gene members (Supplementary Table S2B).

### 2.4. The Identification of Specific LY75-Interaction Proteins Supports the LY75 Role in Modulation of Wnt/β-Catenin Pathway Activity

**Figure 5:**
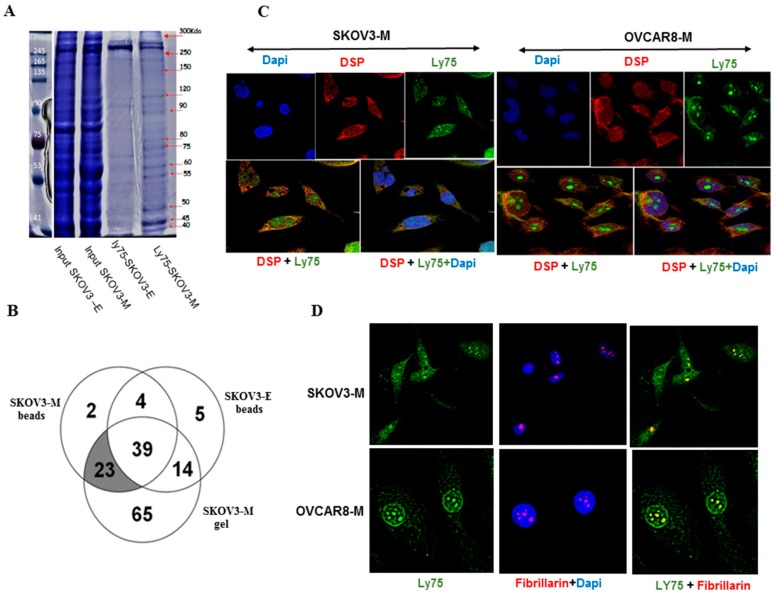
Identification of LY75 interaction partners. (**A**) Gel electrophoresis (Coomassie blue-stained) of anti-LY75 immunoprecipitated protein fractions in SKOV3-E and SKOV-M cells. Bands of interest are indicated by arrows. (**B**) Venn diagram analysis of LY75-interaction proteins identified by LC-MS-MS in SKOV3-M cells, either eluted directly from beads (SKOV3-M beads), or gel-extracted (SKOV3-M gel), compared to nonspecific LY75 interaction proteins extracted from the SKOV3-E (LY75 KD) cells. (**C**) Immunofluorescence analysis of LY75 and DSP cellular co-localization in SKOV3-M and OVCAR8-M cells. The green label is also indicative for a specific nuclear localization of LY75 in both epithelial ovarian cancer (EOC) cell lines. (**D**) Immunofluorescence analysis of LY75 and fibrillarin nucleolar co-localization in SKOV3-M and OVCAR8-M cells.

We further proceeded with the identification of LY75-interaction proteins, as whole cell lysates of SKOV3-M (parental) cells and Ly75 KD SKOV3-E cells were anti-LY75 immunoprecipitated using streptavidin beads, and the resulting peptides were analyzed by liquid chromatography–mass spectrometry (LC-MS/MS). Alternatively, and following the repetition of the same experimental procedure, immunoprecipitated proteins were separated by electrophoresis and specific bands (present in the SKOV3-M fraction and absent in the SKOV3-E fraction) were gel-eluted and subjected to LC-MS/MS (Figure 5A). Venn diagram analysis of the common immunoprecipitated proteins in SKOV3-M (beads) and SKOV3-M (gel), as compared to those in SKOV3-E cells (beads), led to the identification of 23 LY75 specific interaction partners (Figure 5B and Table 2). As shown in Table 2, most LY75 interacting partners (including ACTN4, DNM2, KRT7, KRT8, KRT18, KRT19, SPTAN1, PLEC) were previously known to be involved in actin cytoskeleton remodeling and/or cell trafficking. Importantly, three of the newly identified LY75 partners (DSP, AHNAK, and SPTBN1) were formerly characterized as repressors of the Wnt/β-catenin pathway [17,18,19]. Moreover, immunofluorescence analysis was confirmative of cellular membrane and cytoplasmic co-localization of LY75 and DSP (Figure 5C).

Interestingly, seven nucleolar proteins (including RRBP1, MVP, HSD17B4, HNRNPM, LMNA, KHDRBS1, and RPL6) were among the proteins identified as LY75 interacting partners (Table 2), suggesting for a possible LY75 nucleolar localization. This was further confirmed by immunofluorescence-mediated co-localization analysis of LY75 and the nucleolar protein fibrillarin (Figure 5D).

## 3. Discussion

Numerous studies have previously suggested that EMT induction in cancer is accompanied by a dynamic reprogramming of the epigenome, including DNA methylation alterations, aberrant expression of noncoding RNAs, and post-translational histone modifications. EMT-related epigenetic changes have been described in many cancer types, including EOC [11]; however, the implications of the different epigenetic regulatory mechanisms in EMT-mediated cancer initiation and progression remain largely unexplored [12].

Using an epigenomic approach (methylated DNA immunoprecipitation coupled to CpG island tiling arrays), we have previously shown that DNA hypermethylation occurs in all (comprising less-invasive and early) stages of ovarian tumorigenesis, while advanced disease is exclusively associated with DNA hypomethylation of a number of oncogenes, implicated in EOC progression and invasion/metastasis [20], including genes (LY75, GRHL2, Hic-5, and RUNX1), implicated in EMT regulation [7,21,22,23,24]. In this study, we used the RRBS technology for a comprehensive analysis of DNA methylation changes during LY75-mediated EMT alterations in EOC cells. We initially compared the DNA methylation patterns of SKOV3 control (SKOV3-M) versus LY75-KD SKOV3 (SKOV3-E) cells, using three different experimental combinations, as shown in Table 1B. In each experimental condition, more than 10,000 genes displayed DMRs in their exons and promoter regions, as 6666 common genes were identified under these selection criteria as hypo- or hypermethylated in the SKOV3-E clones (Figure 1A). Based on the 6666 gene list, we further proceeded with a more stringent selection of genes displaying rather high degree (>70%) of methylation in their promoter/exon I regions and previously shown to be implicated in cancer-related EMT signaling. Our stringent selection retained 45 genes (see Supplementary Table S1E), as the DNA methylation status of the promoter regions of most of these genes was further validated by an alternative approach (BSP sequencing). Ten genes were finally selected, whose promoter/exon I DNA methylation patterns completely coincided with their mRNA and protein expression levels. Indeed, HOOK1, RAMP2, JMJD8, and WNT3 appeared hypomethylated and overexpressed (both on mRNA and protein levels) in SKOV3-M cells, whereas EVX2 CLDN5, KLF4, APC2, CEND1, and IFFO1 demonstrated hypomethylated profiles and strong mRNA/protein expression in SKOV3-E cells. These 10 genes also displayed quite similar mRNA and protein expression patterns in OVCAR8-M and OVCAR8-E cells (the latter generated upon shRNA-mediated LY75 KD in OVCAR8 EOC cells; see Supplementary Figure S1A–C). Consecutive analysis of the literature data was indicative for their involvement in carcinogenesis, as some of these genes displayed altered DNA methylation profiles in different cancers. Accordingly, Hook1 (Hook microtubule tethering protein1) represents a microtubule-binding protein involved in microtubule cytoskeleton dynamics, endocytic trafficking, and cell differentiation [25]. It was recently reported that Hook1 inhibits malignancy and EMT in several cancer types, including hepatocellular carcinoma, thyroid cancer and non-small-cell lung cancer [26,27,28]. Ramp2 (receptor activity-modifying protein-2) encodes a family member of single-transmembrane-domain proteins called RAMPs, which transport the calcitonin-receptor-like receptor to the plasma membrane. A specific combination of RAMP members and calcitonin-receptor-like receptor defines Ramp2 ligand affinity for either calcitonin or adrenomedullin [29]. A dual role of Ramp2 has been described in different cancer types; thus Ramp2 and its ligand adrenomedullin were shown to be overexpressed and to promote vascularization and metastasis in human colon cancer [30], while RAMP2 expression has been suppressed by promoter hypermethylation in lung cancer, and ectopic expression of RAMP2 directed apoptosis and inhibited lung cancer cell growth [31]. Similarly, the literature data for Jmjd8 implication in carcinogenesis are rather controversial. Jmjd8 belongs to the family of JmjC domain-redox enzymes that catalyze protein hydroxylation or demethylation [32], as a role of Jmjd8 in regulating cellular metabolism and angiogenesis has been recently reported [33]. An oncogenic role of Jmjd8 as a positive regulator of TNF-induced NF-κB signaling in colorectal cancer has been described [34,35]; however, a recent study demonstrated that JMJD8 knockdown promotes cell proliferation and double-strand base (DSB) repair in lung and bone cancer cells, suggesting that Jmjd8 could represent a potential target for more effective tumor radio- and chemotherapies [36]. As a member of Wnt/β-catenin family, an oncogenic role for Wnt3 has been reported in different cancer types, including colorectal, gastric, breast, liver, and lung cancer [37,38,39,40,41]; yet a potential tumor-suppressor role for Wnt3 has been described in chronic lymphocytic leukemia, where decreased Wnt3 expression is associated with disease progression and worse prognosis [42]. Interestingly, Wnt3 levels were found to be strongly reduced in malignant ovarian tissues compared to normal ovarian tissues [43]. EVX2 (even-skipped homeobox 2) was previously shown to be implicated in vertebrate spinal cord interneuron development [44]. This gene was found highly methylated in lung cancer, where a role for EVX2 as a methylation biomarker for early detection of the disease has been suggested [45]. Claudin5 (CLDN5) is a member of claudin gene family encoding proteins implicated in tight junction formation and function [46]. Cldn5 expression is frequently altered in different human cancers, as CLDN5 gene was found to be downregulated in colorectal, liver, and lung cancer and glioma [47,48,49,50], suggestive for a potential tumor-suppressor role of CLDN5 in these cancer types. However, CLDN5 has been described as an oncogene in breast, pancreatic, and esophageal cancers [51,52,53]. CLDN5 was also found to be highly expressed in malignant EOC tumors compared to benign EOC tumors [54]. Moreover, Cldn5 overexpression correlated with aggressive behavior in serous ovarian adenocarcinoma [55] and was shown to be involved in the malignant transformation of borderline mucinous EOC tumors [56]. Interestingly, CLDN5 was also found to be aberrantly methylated in pancreatic ductal adenocarcinomas [57]. Klf4 (Krüppel-like factor 4) is a zinc-finger-containing transcription factor implicated in regulating cellular growth, proliferation, differentiation, apoptosis, and cell cycle arrest [58]. Recently, a role of KLF4 was reported in inducing pluripotent stem cells and also maintaining the stemness of cancer stem cells [59]. Thus, KLF4 can act as a tumor suppressor or oncogene in different cancer types, largely depending on the cellular context, chromatin structure, cell cycle regulation, and expression patterns of other genes, including specific oncogenic drivers [60]. KLF4 expression was also shown to be frequently mediated through epigenetic or post-transcriptional mechanisms [61]. Moreover, a controversial role of KLF4 in regulating EMT in gastrointestinal cancer has been described [62,63], as currently, the mechanisms underlying its controversial role in this cancer type remain undefined. KLF4 has been characterized as a tumor-suppressor in EOC, as KLF4 downregulation correlated with accelerated EOC proliferation, invasion, and migration and poor patient survival [64,65]. Accordingly, the KLF4 overexpression significantly reduced the metastatic potential of EOC cells by inhibiting the TGFβ-induced EMT [66] and sensitized EOC cells to chemotherapy drugs [65]. Apc2 (adenomatous polyposis coli 2) promotes the assembly of a multiprotein β-catenin destruction complex which results in negative regulation of the Wnt/β-catenin signaling pathway [67]. This protein functions as a tumor suppressor in different cancer types, including prostate, colorectal, and lung cancers, osteosarcoma, retinoblastoma, and glioma [68,69,70,71,72,73], where the APC2 gene expression was frequently found to be inhibited by hypermethylation [72,74,75]. Similarly, a critical role of APC2 in suppressing EOC progression has been demonstrated [76], as APC2 silencing promoted EOC cell proliferation [77] and was associated with decreased overall survival in EOC patients following intraperitoneal chemotherapy [78]. CEND1 (cell cycle exit and neuronal differentiation protein 1) plays an important role in neuronal differentiation by modulating cell cycle progression/exit or apoptosis of neuronal progenitors [79]. CEND1 was shown to suppress cell proliferation via modulating the cyclin D1 pathway, which is linked to its potential tumor suppressor functions, associated with proliferation inhibition of neuroblastoma cells [80]. CEND1 was found to be epigenetically suppressed by methylation in invasive breast carcinoma, also suggestive for a potential tumor suppressor role in this cancer type [81]. IFFO1 (intermediate filament family orphan 1) is a member of the intermediate filament family which includes essential components of the cyto- and nucleoskeleton [82]. Recently, a potential role of IFFO1 in suppressing chromosome translocations during tumorigenesis has been discovered [83]. IFFO1 was reported to be downregulated by promoter methylation in different cancer types [83]. Accordingly, we and others have identified IFFO1 as a highly methylated gene in EOC [20,84], as the IFFO1 methylation has been proposed as EOC biomarker [84].

All the above described genes, epigenetically modulated during EMT in EOC cells, could represent potential prognostic/diagnostic EOC biomarkers. Further studies are warranted to more completely elucidate the functional implications of these 10 genes in ovarian tumorigenesis.

Moreover, our results suggest that Wnt/β-catenin pathway is the principal EMT-related pathway modulated by LY75 expression in EOC cells. *In silico* analysis of the TCGA data for ovarian cancer was also confirmative for the role of LY75 in modulating the Wnt/β-catenin pathway activity in EOC. Deregulations of the Wnt/β-catenin signaling pathway have been associated with the etiology of different human cancers, including colorectal, prostate, breast and skin cancers [85,86,87,88]. Accordingly, the Wnt/β-catenin signaling has been shown to play critical role in EOC development, including EOC stemness, EMT, progression to malignancy, and therapy resistance (recently reviewed in [89,90]). The β-catenin is the key mediator of this pathway, as in the presence of Wnt ligands, the Frizzled and LRP5/6 receptors prevent the formation of the destruction Axin/Apc2/Gskβ complex, allowing the stabilized β-catenin to translocate to the nucleus and to interact with the TCF/LEF transcription factors, thus modulating the transcription of Wnt downstream target genes, many of which regulate EMT, invasion, and metastasis [89].

The IPA canonical pathway analysis of the 6666-gene list displaying common altered DMRs in their exons/promoter regions upon LY75 KD in SKOV3 cells was strongly indicative for the implication of the Wnt/β-catenin pathway in the EMT-associated DNA methylation variations in this EOC cell line (see Figure 3B and Table A1). This was further confirmed following treatment of EOC cells with the Wnt/β-catenin inhibitor XAV939, as XAV939 treatment induced MET-related phenotype changes in SKOV3-M and OVCAR8-M cells (similar to the LY75-KD effect; see Figure 4A), accompanied with suppression of the β-catenin and N-cadherin expression (see Figure 4B). Analogous changes in cells morphology and β-catenin and N/E cadherin switching were not observed following treatment with TGF-β inhibitor SB431542 (Figure 4B). Consecutive immunofluorescence analyses were also indicative for abundant expression and importantly, nuclear localization of β-catenin in SKOV3-M cells, while a rather weak cytomembrane/cytoplasmic β-catenin expression was observed in SKOV3-E cells, accompanied with strong Axin 1 and Apc2 expression (see Supplementary Figure S2).

Furthermore, our proteomics approach led to the identification of 23 LY75 interaction partners mostly involved in actin cytoskeleton organization and/or in cell–cell interactions, which confirms the implication of LY75 in modulating the EOC cellular phenotype (see Figure 5A). Interestingly, three LY75 interaction partners, including spectrin beta chain (SPTBN1), desmoplakin (DSP) and neuroblast differentiation-associated protein (AHNAK) were previously shown to be associated with suppression of the Wnt/β-catenin signaling. Indeed, SPTBN1 has been found to potentiate the expression of the Wnt inhibitor kallistatin in liver cancer [19]. Similarly, DSP was shown to enhance the expression of plakoglobin (also known as γ-catenin) in human lung cancer, which is associated with the suppression of β-catenin [17]. AHNAK has been described as a tumor suppressor in breast cancer that negatively regulates both the AKT/MAPK and Wnt/β-catenin signaling pathways [18]. Thus, our data suggest a possible role of LY75 in maintaining active Wnt/β-catenin signaling in EOC cells by sequestering some of its inhibitors.

Remarkably, seven of the LY75 interaction partners identified were previously recognized as nucleolar proteins [91], and further co-localization immunofluorescence analysis of LY75 and the nucleolar protein fibrillarin was confirmative for a possible LY75 nucleolar localization in EOC cells. Thus, our data suggest a putative LY75 implication in some of the nucleolus functions that mainly include transcription regulation, rRNAs processing, and their subsequent assembly into ribosomal subunits. However, further studies are needed to understand the exact role of LY75 in the nucleolus.

In conclusion, we have shown that LY75-modulated EMT changes directed numerous DNA methylation alterations in EOC cells, as our experimental conditions resulted in the identification of 6666 genes displaying common altered DMRs in their exons/promoter regions. Ten genes were consecutively identified with significant methylation alterations in their promoter regions, which corresponded with their expression levels in EOC cells, suggesting their further investigation as potential EOC biomarkers/therapeutic targets. Moreover, LY75-mediated EMT alterations in EOC cells were predominantly associated with epigenetic regulation of members of the Wnt/β-catenin pathway. We further demonstrated that LY75 supports an active status of the Wnt/β-catenin pathway in EOC cells, while the LY75 depletion predominantly directs the pathway suppression. Though, a possible back-loop regulation of the LY75 expression via the Wnt/β-catenin signaling cannot be excluded. Our data are also indicative for a possible LY75 nucleolar localization; however, the role of LY75 in the nucleolus needs to be further elucidated.

A comprehensive understanding of molecular processes and the chronology of events between DNA methylation and the signaling pathways triggering EMT in EOC could arrange for the development of more effective (including epigenetic) treatment strategies for this deadly disease.

## 4. Materials and Methods

### 4.1. Cell Cultures

The SKOV3 and OVCAR8 EOC cell lines were purchased from American Tissue Type Collection (Manassas, VA, USA). Cells were maintained in RPMI-1640 medium supplemented with 10% (v/v) fetal bovine serum (Hyclone, Logan, UT, USA) and cultured in a humidified incubator with 5% CO_2_ at 37 °C as previously described [7]. In some experimental conditions, SKOV3 and OVCAR8 cells were treated for 24 h with the Wnt/β-catenin inhibitor XAV939 (Sigma-Aldrich, Oakville, ON, Canada) and for 5 days with the TGF-β inhibitor SB431542 (Reagents Direct, Encinitas, CA, USA), as both inhibitors were applied at a final concentration of 20 µM. The corresponding SKOV3 and OVCAR8 control cells were treated with 0.1% DMSO.

### 4.2. Reduced Representation Bisulfite Sequencing (RRBS) Analysis

For RRBS analysis, ~10 µg of genomic DNA was extracted from different SKOV3-M and SKOV3-E clones using the Blood & Cell culture DNA Mini Kit (Qiagen, Montreal, PQ, Canada). RRBS analysis was performed on a service basis by the company Diagenode Inc. (https://www.diagenode.com/en/p/rrbs-service). The comparisons between the RRBS datasets were carried out using the R package methylKit, using the hg19 refGene and CpG island annotation from the UCSC GenomeBrowser database (http://genome.ucsc.edu). Differentially methylated CpGs, as well as differentially methylated regions (DMRs) were identified with a >25% methylation difference and an adjusted *p*-value < 0.05 (the latter with a window size of 1000 bp, since this has been found to include the majority of DMRs [92]). The DMRs were annotated using the UCSC RefSeq tracks (hg19) to further analyze CpG sites included in genes’ exons and promoter regions (window size of 25 bp; promoter regions spanning around 2 kb upstream and downstream of the transcriptional start site). All RRBS sequencing and processed data were deposited in the Gene Expression Omnibus (GEO) with accession number GSE142310.

### 4.3. Bisulfite Sequencing PCR (BSP) Analysis

BSP analysis was performed, as previously described [20]. BSP primer selection was performed using the Methyl Primer Express Software v1.0 (Applied Biosystems, Waltham, MA, USA); all primers are listed in Table A3. PCR was done for 40 cycles (94 °C, 45 s; 54–60 °C, 45 s; 72 °C, 45 s), as shown before [20]. PCR products were sent for dideoxy-sequencing analysis at the Genomics Analysis Platform at Laval University (http://www.bioinfo.ulaval.ca/seq/en/).

### 4.4. Short Hairpin RNA (shRNA)- ediated LY75 Knockdown in EOC Cells

The shRNA-mediated LY75 knockdown in EOC cells was done, as previously described [7]. Briefly, two LY75 shRNAs cloned into the pLKO.1-puro vector (targeting the LY75 mRNA sequences 5′-GCCCUAAUACUCAACCUCCAA-3′ and 5′-UCCCGTCUUACAUAUUCAUCAA-3′) were retrieved from the Sigma Mission TRC human 1.5 shRNA library (clone numbers TRCN0000057363 and TRCN0000057364). Viral supernatants were generated by transfecting 293T cells with the shRNA constructs and the packaging vectors psPAX2 and pMD2.G (Addgene, Cambridge, MA, USA). The high-titer lentiviral supernatants in the presence of 8 µg/mL polybrene were used to infect SKOV3 and OVCAR8 cells. Two days later, infected cells were treated with puromycin (2 µg/mL) for the selection of stably-transduced clones. The pLKO.1-puro vector encoding a scramble sequence not matching any mammalian sequence was used for the generation of mock-transduced (control) clones. Stable clones with inhibited LY75 expression were evaluated and validated by quantitative RT-PCR and Western blot.

### 4.5. Western Blot Analysis

Western blot analyses were performed as previously described [7]. List of antibodies used: anti-LY75 (Abcam, Branford, CT, USA and Santa Cruz Biotechnology Dallas, TX, USA), anti-Snail1, anti-FN1, anti-E-cad, anti-EpCAM, anti-AXIN1, anti-WNT3, anti-EVX2, anti-IFFO1, anti-CLDN5, anti-HOOK1, anti-JMJD8, anti-RAMP2, anti-KLF-4, anti-β-actin antibodies (Santa Cruz Biotechnology, Dallas, TX, USA) and anti-Twist1, anti-E-cadherin, anti-N-cadherin, anti-APC2, and anti-Cend1 antibodies (Abcam Branford, CT, USA).

### 4.6. Quantitative PCR (qPCR)

Quantitative PCR was performed as previously described [7]. Briefly, total RNA was extracted by the RNeasy Plus Mini Kit (Qiagen, Montreal, PQ, Canada) and RNA was reverse transcribed into cDNA using Superscript III transcriptase, according to the manufacturer’s protocol (Invitrogen; Thermo Fisher Scientific, Waltham, MA, USA). RT-qPCR was performed using the SYBR Green PCR Master Mix (Applied Biosystems; Thermo Fisher Scientific Waltham, MA, USA) on ROTOR GENE real-time PCR machine (Corbett research, Corbett Robotics, Brisbane, Australia). Relative quantification of RNA expression was calculated using the 2^−ΔΔCq^ method [93]. The 18S ribosomal gene was used as an internal standard. Each sample was tested in triplicate. Primers were designed as previously shown [7]; all primers for qPCR are listed in Table A2.

### 4.7. Immunoprecipitation and Consecutive Mass Spectrometry (MS) Analysis

For immunoprecipitation, SKOV3-M and SKOV3-E cells were lysed in 1 mL cell RIPA lysis buffer. Following lysis, 500 μg of proteins were incubated with 2 μg of the LY75 antibody (Santa Cruz Biotechnology, Dallas, TX, USA) for 4 h at room temperature and overnight at 4 °C upon gentle rotation, before being incubated with 40 μL Dynabeads Protein G (Invitrogen, Waltham, MA, USA) at 4 °C for 2 h under rotation. Beads were digested with trypsin and the resulting peptides were analyzed by LC-MS/MS. The same immunoprecipitation experiment was repeated once again; however, this time the beads were consecutively resuspended in 40 µL SDS sample buffer and boiled for 10 min; the supernatant was analyzed by electrophoresis and stained by Coomassie blue. Gel bands of interest of the SKOV3-M peptide fraction were digested with trypsin, and the resulting peptides were analyzed by LC-MS/MS. Protein digestion and MS analyses were performed at the Proteomics Platform of the CHU de Québec Research Center (Quebec, PQ, Canada), using the Ekspert NanoLC425 HPLC system (Eksigent technologies Dublin, CA, USA) coupled to a 5600+ mass spectrometer (Sciex, Framingham, MA, USA) equipped with a nanoelectrospray ion source, as previously described [94]. MGF peak list files were consecutively created using Protein Pilot version 4.5 software (Sciex, Framingham, MA, USA). MGF sample files were then analyzed using Mascot (Matrix Science, London, UK; version 2.5.1). Mascot was set up to search the contaminants_thegpm_20170713.fasta; CP_HomoSapiens_9606_CI_20170329_2 database (unknown version, 92,988 entries) assuming the digestion enzyme trypsin. Mascot was searched with a fragment ion mass tolerance of 0.100 Da and a parent ion tolerance of 0.100 Da. Carbamidomethyl of cysteine was specified in Mascot as a fixed modification. Deamidated of asparagine and glutamine and oxidation of methionine were specified in Mascot as variable modifications. Scaffold software (version Scaffold_4.8.4, Proteome Software Inc., Portland, OR, USA) was used to validate MS/MS-based peptide and protein identifications. An FDR less than 1.0% for peptide and protein was used. Proteins that contained similar peptides and could not be differentiated based on MS/MS analysis alone were grouped to satisfy the principles of parsimony.

### 4.8. Immunofluorescence

Cells were plated on poly-d-lysine coated slides (Sigma Aldrich Oakville, ON, Canada), then fixed in 4% paraformaldehyde and permeabilized with 1 x PBS–0.2% Triton X-100. After blocking, cells were incubated with different primary antibodies, including anti-β-catenin (Santa-Cruz Biotechnology Dallas, TX, USA), anti-LY75 (Santa-Cruz Biotechnology Dallas, TX, USA and Abcam, Branford, CT, USA), anti-APC2 (Abcam, Branford, CT, USA), anti-Axin1 (Santa-Cruz Biotechnology Dallas, TX, USA), and anti-DSP (Santa-Cruz Biotechnology Dallas, TX, USA), and subsequently incubated with secondary antibodies, including rhodamine-linked goat-anti-mouse IgG1 (Santa Cruz Biotechnology Dallas, TX, USA) or Alexa Fluor 488-labeled goat anti-rabbit antibody (Abcam, Branford, CT, USA). Cells were finally stained with 4′,6-diamidino-2-phenylindole (DAPI). Images were captured using a Zeiss LSM 700 confocal microscope (Carl Zeiss Meditec AG Jena, Germany).

## Figures and Tables

**Table 1:** Reduced representation bisulfite sequencing (RRBS) analysis of altered DNA methylation patterns upon Ly75-mediated epithelial–mesenchymal transition (EMT) variations in SKOV3 cells.

**A. Description of the SKOV3 Clones Used (as Described in [14])**	**Phenotype**
shC-S: control shRNA expressed in SKOV3 cells	Mesenchymal (M)
sh-LY75-S: shRNA-mediated LY75-KD in SKOV3 cells	Epithelial (E)
LY75-KO-S: CRISPR/Cas9-mediated Ly75 KO in SKOV3 cells	Epithelial (E)
LY75-shR-S: sh-resistant-Ly75 cDNA expressed in sh-LY75-S cells	Mesenchymal (M)
**B. Experimental Comparison Combinations Used for RRBS Analysis**	**Number of Differently Methylated Regions (Hypo + Hyper) Identified in Exons and Promoter Regions of Different Genes**
shC-S vs. sh-LY75-S (M vs. E)	10,722
LY75-R-S vs. LY75-KO-S (M vs. E)	11,014
(mix: shC-S + LY75-R-S) vs. (mix: sh-LY75-S + LY5-KO-S) (M vs. E)	9105

**Table 2:** Ly75 partners identified by the immunoprecipitation experiments.

Ly75 Partners Identified by Scaffold	M Weight
AHNAK: Neuroblast differentiation-associated protein	629 kDa
PLEC: Isoform 4 of Plectin	516 kDa
DSP: Desmoplakin	332 kDa
SPTAN1: Spectrin alpha chain, nonerythrocytic	285 kDa
SPTBN1: Spectrin beta chain	275 kDa
CAD protein	243 kDa
RRBP1: Ribosome-binding protein 1	152 kDa
ACTN4: Alpha-actinin-4	105 kDa
MVP: Major vault protein	99 kDa
DNM2: Isoform 2 of Dynamin-2	98 kDa
HSD17B4: Peroxisomal multifunctional enzyme type 2	80 kDa
HNRNPM: Heterogeneous nuclear ribonucleoprotein M	78 kDa
LMNA: Prelamin-A/C	74 kDa
PABPC1: Polyadenylate-binding protein 1	71 kDa
SEPT9: Septin-9	65 kDa
KRT8: Keratin, type II cytoskeletal 8	54 kDa
SPATS2L: Isoform 2 of SPATS2-like protein	54 kDa
KRT7: Keratin, type II cytoskeletal 7	51 kDa
KRT18: Keratin, type I cytoskeletal 18	48 kDa
KHDRBS1: KH domain-containing, RNA-binding, signal transduction-associated protein 1	48 kDa
KRT19: Keratin, type I cytoskeletal 19	44 kDa
RPL6: 60S ribosomal protein L6	33 kDa

## Supplementary Materials

Supplementary materials can be found at https://www.mdpi.com/1422-0067/21/5/1848/s1.

## Abbreviations

EOC	Epithelia ovarian cancer
EMT	Epithelial–mesenchymal transition
MET	Mesenchymal–epithelial transition
ncRNA	Noncoding RNA
RRBS	Reduced Representation Bisulfite Sequencing
IPA	Ingenuity Pathway Analysis
DMRs	differentially methylated regions
IGV	Integrative Genomic Viewer
BSP	Bisulfite-sequencing PCR
shRNA	Short hairpin RNA
q-PCR	Quantitative PCR
LC-MS/MS	liquid chromatography–mass spectrometry

## Appendix A

**Table A1:** List of genes displaying EMT-related DNA methylation alterations upon IPA analysis of the EMT, Wnt/β-cat and TGF-β canonical pathways, including common genes between EMT/Wnt/β-cat or EMT/TGF-β canonical pathways.

EMT Genes		Wnt/β-Cat Genes		TGF-β Genes		Common EMT/Wnt/β-Cat Genes	Common EMT/TGF-β Genes	
WNT3	PDGFD	CDKN2A	EP300	INHA		WNT3	SMAD3	
AXIN1	NOTCH1	WNT3	AKT1	BMP4		AXIN1	HRAS	
PIK3R1	WNT1	AXIN1	JUN	SMAD3		WNT6	MAP2K2	
SMAD3	FGF5	PPP2R5B	CDH3	SKI		WNT7B	SOS1	
HRAS	RELA	TLE1	UBA52	HRAS		WNT4	GRB2	
PARD6G	FZD10	WNT6	DKK3	BMPR1B		WNT5B		
WNT6	FGF8	SOX13	TGFB2	MAPK13		AKT2		
PIK3R4	TWIST1	MYC	SOX14	MAPK11		WNT9A		
FGFR3	KLB	TGFB1	CSNK2B	TLX2		FZD9		
FOXC2	HNF1A	PPM1J	SOX7	TGIF1		TCF3		
MAP2K2	WNT2	WNT7B	LRP5	EP300		CDH1		
TGFB1	mir34	RARA	WNT9B	BCL2		CDH12		
WNT7B	FGF13	WNT4	CSNK1G3	JUN		FZD6		
FGF12	FGF4	MAP4K1	WNT2B	MAP2K2		LEF1		
WNT4	AKT1	WNT5B	DVL1	TGFB1		WNT1		
IRS2	KL	AXIN2	DKKL1	SOS1		FZD10		
WNT5B	SOS1	AKT2	TCF7L1	TGFB2		HNF1A		
FGF19	TGFB2	CSNK1G2	SOX11	MAP4K1		WNT2		
PIK3C2B	FGF23	WNT9A	FZD8	VDR		AKT1		
AKT2	PIK3R2	CREBBP	WNT3A	HNF4A		WNT9B		
TWIST2	FGF3	CSNK1D	WNT10A	RUNX3		WNT2B		
JAG2	EGFR	FZD9	SOX6	GRB2		DVL1		
WNT9A	MAP2K7	TCF3	APC2	FOXH1		TCF7L1		
TYK2	ESRP2	ACVR1B	NR5A2	CREBBP		FZD8		
FGFR2	WNT9B	CDH1	PPP2R5E	SMAD6		WNT3A		
FZD9	GRB2	CDH12	SOX15	SMAD7		WNT10A		
MMP2	EGR1	TLE3	WNT11	MAPK9		WNT11		
NFKB2	WNT2B	FZD6	LRP1	PITX2				
ZEB1	DVL1	LEF1	SOX3	MAPK12				
TCF3	FGF14	SOX8		ACVR1B				
TLR9	TCF7L1	SFRP1		INHBB				
MET	FGF21	WNT1		TRAF6				
CDH1	FZD8	FZD10		NKX2-5				
CDH12	WNT3A	SFRP2		FOS				
IRS1	WNT10A	FRZB		ZNF423				
mir-192	ZEB2	SOX1		IRF7				
FZD6	FGF20	FRAT1		SMURF2				
LEF1	FGF11	MARK2		BMP7				
FGFRL1	RBPJ	HNF1A		PMEPA1				
WNT11	JAK3	WNT2						

**Table A2:** Primers used for quantitative PCR (qPCR).

Gene	q-PCR Primers	Sequence (5′->3′)
HOOK1 (NM_015888)	Forward	AGTGAGTTGACACCCTGTGG
	Reverse	TGGTATATGTACTCAAGCCTCCC
	Product length (pb)	103
JMJD8 (NM_001005920)	Forward	TCATCACCCTCGTGGTTTCG
	Reverse	ATCTGGCCAGGTCCATCTCT
	Product length (pb)	83
RAMP2 (NM_005854)	Forward	GCACGAGCTTCTCAACAACC
	Reverse	CAACCCTGGCTTCCATTCCC
	Product length (pb)	134
KRT7 (NM_005556)	Forward	ATTCCACTGGTGGCAGTAGC
	Reverse	TGGAGAAGCTCAGGGCATTG
	Product length (pb)	83
APC2 (NM_001351273)	Forward	GCTCCGACAGCATTACCTCA
	Reverse	AGACCCGGTACAGAAACGTG
	Product length (pb)	115
CEND1 (NM_016564)	Forward	TACATACGCCCCCAACACAC
	Reverse	TCTTTCTGCCCTGGAGGTTG
	Product length (pb)	92
CLDN5 (NM_001130861)	Forward	GGATTTCGCTTCCCCTCCAA
	Reverse	GTACACATCTTCCGGTGGGG
	Product length (pb)	119
EVX2 (NM_001080458)	Forward	GGGAGAACTATGTGTCGCGG
	Reverse	CGGTTCTGGAACCACACCTT
	Product length (pb)	91
KLF4 (NM_004235.6)	Forward	AGAACAGATGGGGTCTGTGAC
	Reverse	TCCACAACTTCCAGTCACCC
	Product length (pb)	106
WNT3 (NM_030753)	Forward	ATCTACGACGTGCACACCTG
	Reverse	TGCTTCCCATGAGACTTCGC
	Product length (pb)	95
18S (NR_003278)	Forward	AACCCGTTGAACCCCATT
	Reverse	CCATCCAATCGGTAGTAGCG
	Product length (pb)	119

**Table A3:** Primers used for BSP analysis.

Genes	Primers	Outer PCR-Sequence (5′->3′)	Inner PCR-Sequence (5′->3′)
HOOK1 (NM_015888)	Forward	GGTTTAGGTGTTTGGTAGYG 3	GGTTTAGGTGTTTGGTAGYG
	Reverse	ACCCAAATCATAAAACTATCRC	ACCRACTCTCCTCCAAAAA
	Product length (pb)	318	204
JMJD8 (NM_001005920)	Forward	ATGAAGTGGTTGGAAGGTAGTT	GGAGGTTGGAATTTGAGATTT
	Reverse	CRAAAATAACCTCCTTTAACCC	CRAAAATAACCTCCTTTAACCC
	Product length (pb)	367	231
RAMP2 (NM_005854)	Forward	TTTTTTTTTTGTTGGGYG	TTTTTTTTTTGTTGGGYG
	Reverse	CTTATCACTCACACCCAAACC	CCCCTCATCTCTAACCAACTT
	Product length (pb)	318	291
KRT7 (NM_005556)	Forward	TTTGGATTGAAAGTTTGG	TGGTAGTAGAGAAAGGTGGTT
	Reverse	AACCCCCATAAACAAAAC	AACCCCCATAAACAAAAC
	Product length (pb)	396	325
APC2 (NM_001351273)	Forward	TTGGTTGTTGTTATGGTATTAGT	TTGGTTGTTGTTATGGTATTAGT
	Reverse	AACTCAATTTCCCCTCCAA	CCTCCAACTCCCACTCTAA
	Product length (pb)	447	435
CEND1 (NM_016564)	Forward	AGTAGTGTATTGTGGGAAATTTT	AGTAGTGTATTGTGGGAAATTTT
	Reverse	ACTACTACCACCTCCCAAA	CCCAATAACCTTCAAAACC
	Product length (pb)	391	191
CLDN5 (NM_001130861)	Forward	GTAAATTTTGGTTAGGGAAGTG	GTAAATTTTGGTTAGGGAAGTG
	Reverse	CACCTCCTAAATCTACCAACTC	ACCAATCACAAAACCTCTAACAA
	Product length (pb)	436	310
EVX2 (NM_001080458)	Forward	GGGTTATTGTGATATTTTTAAGAA	TGGAGAGAGGGTTGTATAGTT
	Reverse	ATTACCTTTACCATTATTTTCCTT	ATTACCTTTACCATTATTTTCCTT
	Product length (pb)	463	398
KLF4 (NM_004235.6)	Forward	ATTTTTTGGATTTGGATTTTATT	ATTTTTTGGATTTGGATTTTATT
	Reverse	AAATATACACCRAATCCAATTC	AAACRAACTCCCTACCATA
	Product length (pb)	339	266
WNT3 (NM_030753)	Forward	AGGAAATGTAAAGGTAGTAGGAG	AGGAAATGTAAAGGTAGTAGGAG
	Reverse	AAAAACACAAAAATATTTCCAA	ACAAAAATATTTCCAAAAACCC
	Product length (pb)	286	280
