# Supplementary material for: LY75 Ablation Mediates Mesenchymal-Epithelial Transition (MET) in Epithelial Ovarian Cancer (EOC) Cells Associated with DNA Methylation Alterations and Suppression of the Wnt/β-Catenin Pathway
Source: Int J Mol Sci. 2020 Mar 7;21(5):1848. doi: 10.3390/ijms21051848 (PMC7084525; doi:10.3390/ijms21051848)
Supplement: Supplementary file 1 [file ijms-21-01848-s001.zip › Supplementary Figures.pdf]

Supplementary Figures

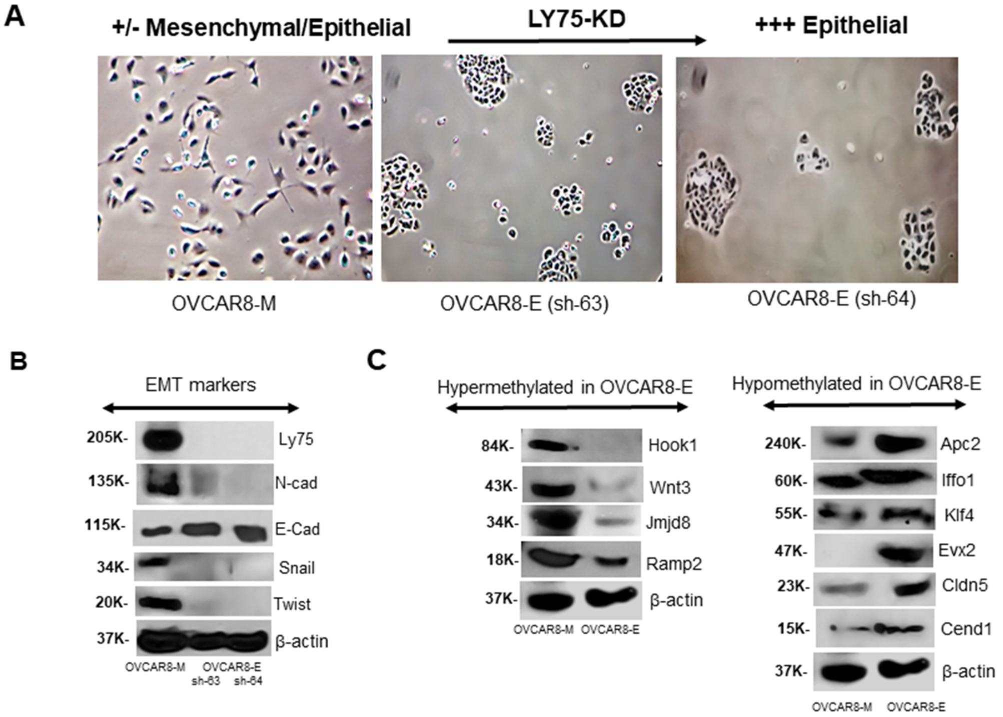

Figure S1. (**A**) Representative phase-contrast images of OVCAR8-M cells (sh-control) and OVCAR8-E cells (LY75-KD clones: sh-63 and sh-64). (**B**) Western blot analysis of the protein expression levels of different EMT markers in OVCAR8-M and OVCAR8-E cells. (**C**) Western blot analysis of the protein expression levels of the ten RRBS-selected genes in OVCAR8-M and OVCAR8-E cells. β-Actin was used as a loading control.

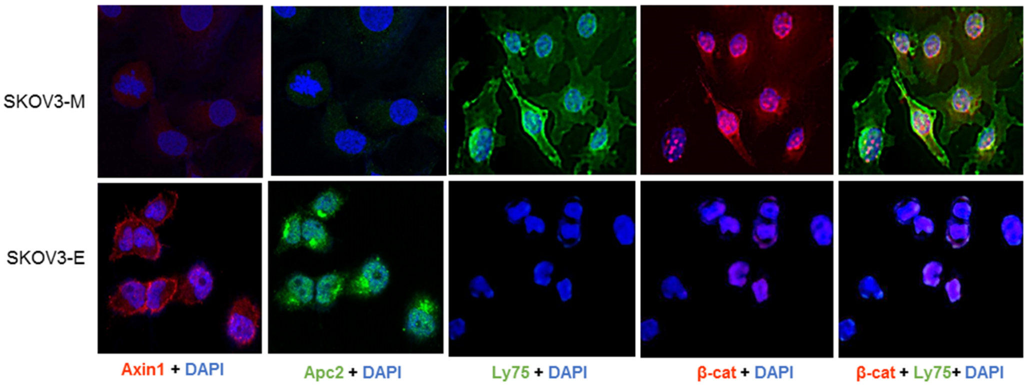

Figure 2S. Immunofluorescence analysis of Axin1, Apc2, β-catenin and Ly75 expression and localization in SKOV3-M and SKOV3-E cells.
