# Supplementary figures and images for: LY75 Ablation Mediates Mesenchymal-Epithelial Transition (MET) in Epithelial Ovarian Cancer (EOC) Cells Associated with DNA Methylation Alterations and Suppression of the Wnt/β-Catenin Pathway
Source: Int J Mol Sci. 2020 Mar 7;21(5):1848. doi: 10.3390/ijms21051848 (PMC7084525; doi:10.3390/ijms21051848)

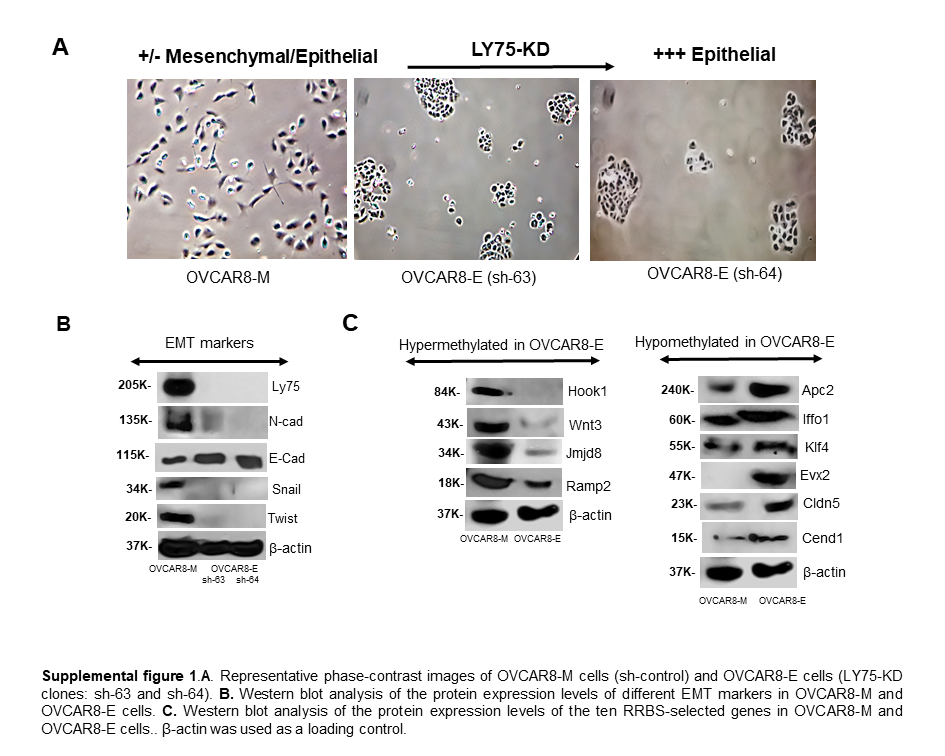

Supplement: Supplementary file 1 [file ijms-21-01848-s001.zip › Supplementary Figure S1.tif]

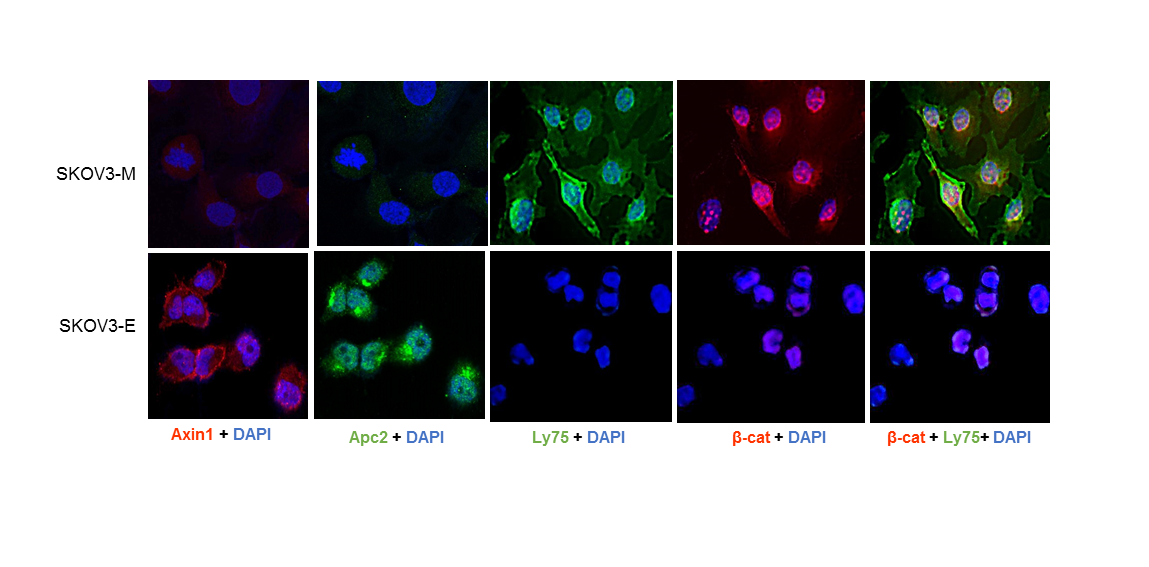

Supplement: Supplementary file 1 [file ijms-21-01848-s001.zip › Supplementary Figure S2.tif]
